# Erythroxylon Coca and Its Alkaloid—Cocaine

**Published:** 1887-08

**Authors:** W. E. Baxter

**Affiliations:** Owenton, Ky.


ARTICLE IV.

### ERYTHROXYLON COCA AND ITS ALKALOID—COCAINE.

BY W. E. BAXTER, OWENTON, KY.

[Read before the Kentucky State Dental Society, June, 1887.]

The coca leaf is the great source of comfort and enjoyment to the Peruvian Indian; it is to him what betel is to the Hindoo, and tobacco to the rest of mankind. But the other stimulants do not possess the invigorating effects that the coca leaf produces. The Peruvian Indians have used this leaf from the most ancient times, and still look upon it with feelings of veneration and superstition. It was sacrificed to the sun in the time of the Incas, the high priest chewing the leaf during the ceremony; and before the arrival of the Spaniards it was used instead of money. After the conquest it was proposed to proscribe its use, because it had been used in the ancient superstitious rites. In 1569 the second Council of Lima condemned the use of coca in South America, because it was a "useless and pernicious leaf," and on account of the belief stated to be entertained by the Indians that the habit of chewing coca gave them strength, "which is an illusion of the devil."

In speaking of the strength the coca gives to those who chew it, Carcilasso de la Vega relates the following anecdote: "I remember a story which I heard in my native land of Peru, of a gentleman of rank and honor, named Rodrigo Pantoja, who traveling from Cuzed to Lima met a poor Spaniard who was going on foot with a little girl aged two years on his back. The man was known to Pantoja, and they thus conversed: 'Why do you go laden thus?' said the knight. The poor man answered that he was unable to hire an Indian to carry the child, and for that reason carried it himself. While he spoke Pantoja looked in his mouth and saw that it was full of coca; and as the Spaniards abominate all that the Indians eat and drink, as though it savored of idolatry, particularly the chewing of coca, which seems to them a low and vile habit, he said: 'It may be as you say, but why do you eat coca, like an Indian, a thing so hateful to Spaniards?' The man answered, 'In truth, my lord, I detest it as much as any one, but necessity obliges me to imitate the Indians and keep coca in my mouth, for I would have you to know that if I did not do so I could not carry this burden, while the coca gives me sufficient strength to endure the fatigue.' Pantoja was astonished to hear this, and told the story wherever he went, and from that time credit was given to the Indians for using coca from necessity, and not from gluttony.

Cocaine, the alkaloid of erythroxylon coca, was discovered by Hieman in 1860. Hieman prepared cocaine by exhausting coca leaves with 85 per cent. of alcohol, containing 1–50 of sulphuric acid, supersaturating the alcoholic solution with lime, then neutralizing carefully with dilute sulphuric acid, separating the precipitated sulphate of calcium and distilling off the alcohol. The residuary liquid is supersaturated with soda, and then shaken repeatedly with ether, which dissolves out the cocaine. On evaporating the etherial solution cocaine remains behind in an amorphous condition, but soon becomes crystalline.

According to Merck, coca contains between 1–5 and 1–50 per cent. of cocaine. The average effective dose of his hydrochlorate in man is stated to be ¾ of a grain.

Schroff, who made the first experiments with cocaine in 1862, observed that it caused fluctuations in the respirations and pulse, and produced mydriasis. Fronmuller, in 1863, found that it had but little effect, in man, in doses of 2–5 to ½ grain, and in a case of attempted suicide 24 grains did not even produce serious results. The cocaine used in those days must have been very impure.

Since I began to use cocaine for extracting, in January, 1885, I have had no occasion to use any other preparation of muriate cocaine than Park, Davis & Co.'s 4 per cent. solution, as I have met with uniform results with this preparation in lessening pain, with few exceptions, since I learned how and where to insert the point of the hypodermic needle; and all depends on this one thing to have good results.

In this time I have used altogether about 150 drachms of the 4 per cent. solution, and the greatest quantity used hypodermically for one person at one time was 1½ drachms, 4 per cent. This was for Mrs. Wm. G. C., of Dallasburg, who was unable at the time to come to the office; hence I was sent for to do the extracting (28 teeth in all) at her home. The lady had suffered greatly with her teeth, the mouth being in a very bad condition, most of the teeth badly abscessed with calcareous deposits on their necks, gums badly swollen and hypersensitive from stagnant circulation. The tissue surrounding the teeth was injected with cocaine until it was thoroughly *bleached*; and when this occurs you may know that the cocaine is of good strength, and has had the proper effect, and you can extract at once. These teeth were taken out without the slightest pain, and although the patient being of a hemorrhagic diathesis, and had head trouble before, from great flow of blood from extracting, we had no trouble whatever from undue bleeding, as the cocaine undoubtedly had a beneficial effect in this direction, and I have often noticed that the flow of blood is not so great when it is used, as it seems to drive the blood from the parts in which it is injected.

Great care should be taken that none of the cocaine should get in the mouth and be swallowed. Cocaine can be taken in the stomach by some without bad results, while I have observed that one drop taken on the tongue and swallowed has caused violent retching, the nausea lasting from one to two hours. This, however, can be relieved almost instantly by dr. doses of Hoffman's anodyne in 3 times amount of water. This can be repeated at intervals of five minutes until sickness is relieved, one dose, though, generally does the work.

Miss Bettie T., aged 16, came to the office on the 5th of last March to have a left superior bicuspid extracted. The patient was predisposed to heart trouble, her mother dropping dead while apparently enjoying the best of health. Miss T. was of a highly nervous temperament. She had suffered with this tooth for some time, and being a chlorotic patient, with this and overwork in the school room she had become much debilitated. Another indication of feebleness and nervousness was the tremulous moist and flabby tongue. I apprehended trouble and watched the patient closely. I injected 10 minims in the gum surrounding the tooth, the reddened gum turning white, whereupon the tooth was taken out, and as the patient told me afterwards without the slightest pain. As soon as the tooth was out the patient "collapsed," pulse and breath ceased. Artificial respiration with nitrate of amyl to the nostrils was resorted to with good results, the patient remaining in a semi-conscious state. She was placed in a recumbent position, there was then a rush of blood to the head; the extremities became cold and numb. The blood would at times leave the head, the patient stop breathing, the jaw become rigid, whereupon artificial respiration would be used, and when the patient would come to again she would beg to let her go to sleep. This should never be allowed. Patient complained of being sick at stomach, palpitation of heart was noticeable, at times, the breathing difficult with sense of suffocation, and then stupor with insensibility, and when the patient would partially rally there would be agitation of limbs and whole body. Hoffman's anodyne, well known as a diffusible stimulant, anodyne and antispasmodic, was given in drachm doses every five minutes. After the first dose there was a noticeable change in the patient's condition; the nausea disappeared, the breathing and pulse became more regular, and after the third dose she was able to leave the office in seemingly better condition than when she came in.

In this case we have several indications pointing to hysteria, and as the patient had a similar *sceance* before from having a tooth extracted without an anæsthetic, I am loth to believe that the trouble was caused by cocaine. I could cite a number of cases in which I have had trouble with patients of a highly nervous temperament without using anæsthetics, and afterwards had the most flattering results from the use of cocaine.

We all know the pain attendant on applying the rubber dam over deep seated cavities, whose margins extend beneath the gums, and especially if the gums are in an unhealthy condition. I now make this operation painless by first *bleaching* the surrounding gum tissue with cocaine, after which the rubber dam, separators, matrices, etc., can be applied with all ease. The addition of muriate of morphia to muriate of cocaine hastens as well as prolongs the anæsthetic effect of cocaine. Add one grain of muriate of morphia to one drachm of distilled water, and this to one dram of four per cent. solution muriate cocaine; 20 drops of this gives the ordinary dose of morphine, one-sixth of a grain; inject 5 minims of this, etc.

I believe the operation of implantation can be made painless in this manner, although Dr. Younger says he has no confidence in cocaine, and this is because he never injected it, but only applied it to the gum surface, and of course it could not be taken up by the capillaries of the surrounding tissues to any great extent, and give the anæsthetic effect desired. As an obtunder for sensitive dentine it has not met with the success desired, but in the painless extirpation of pulps it has proved invaluable.

I am an advocate of the preservation of the natural teeth and their crownless roots, and "preach" (except Sundays) from this text almost every day in the year; but have yet to see the dentist who is so conscientious that he will take nearly all of his time in doing pauper practice. I usually suggest to these patients the advisability of saving their teeth, and then if they can't pay for the operation, and want them extracted, I do it in the easiest manner possible.

It is a wise provision of nature that great pain should attend the operation of extracting teeth—thus scaring the patient into taking better care of these organs—yet the day (as predicted) will never come, when the so-called "murderous forceps" will be laid aside, as we will find it advisable in many cases to extract and to have teeth extracted, and will be most loud in praise of that which will relieve the pain of this much dreaded operation.

Gentlemen, I have taken up too much of your time, and hope you will bear with me in this my first humble effort; as "you have been there yourself," probably, and know what it is to make your "first bow" on paper before such an intelligent and scientific body of men as the Kentucky State Dental Association.—*Dental Register.*

